# Laparoscopic Umbilicus-Sparing Excision of Symptomatic Patent Urachus in Adulthood: Case Report and a Review of the Literature

**DOI:** 10.7759/cureus.64471

**Published:** 2024-07-13

**Authors:** Omar Varela Barraza, Ernesto Dávila Legorreta, Lucero Huerta Hernandez, Antonio Esqueda-Mendoza, Irma L Maldonado Barrios, Mariana Gutiérrez Neri Pérez, Martha E Gutiérrez Brambila

**Affiliations:** 1 Department of General Surgery, Hospital Regional de Alta Especialidad del Bajío, León, MEX; 2 Department of Urology, Hospital Regional de Alta Especialidad del Bajío, León, MEX; 3 Department of Urology, El Hospital Regional de Alta Especialidad de la Península de Yucatán, Mérida, MEX

**Keywords:** umbilicus-sparing, urachal, urachal anomalies, patent urachus, urachal remnant

## Abstract

Urachal abnormalities are infrequent pathologies characterized by the failure of obliteration of the urachal canal, resulting in a persistent fibrous cord. The initial diagnosis approach can be done with ultrasonography; however, computed tomography is considered a standard diagnostic method. The preferred method for relieving symptoms and reducing recurrence is surgical excision. This is a case of a 45-year-old man with recurrent urinary tract infections, suprapubic pain, and umbilical fetid discharge who underwent laparoscopic umbilicus-sparing excision.

## Introduction

The urachus is embryologically a ductal fibrous cord arising from the fetal bladder wall and extends to the allantois, which facilitates the removal of nitrogenous waste through the placenta. During its development, the bladder descends to the fetal pelvis, leading to the obliteration of the urachal lumen, forming a fibromuscular cord-like structure that stretches from the bladder dome surface to the umbilicus behind the abdominal wall in the extraperitoneal space. This band is known as the median umbilical ligament [1].

Congenital obliteration failure results in the following distinct uncommon and often underdiagnosed entities: patent urachus, umbilical-urachal sinus, vesicourachal diverticulum, urachal fistula, and urachal cyst. Up to 80% of the urachal defects disappear after two years of birth; the remaining 20% may persist unrecognized, being discovered during medical imaging tests, or might develop vague stomach pain, urinary tract infections, hematuria, and umbilical discharge [2].

Early diagnosis and optimal treatment for infected urachal abnormalities prevent complications, such as rupture and fistula formation, that may trigger peritonitis, abscess, or sepsis, with the need for urgent surgical intervention. These anomalies have also been associated with an increased risk of urachal and bladder malignancy [3].

Surgical excision of the urachus remnant represents the mainstay of treatment for localized disease, but the appearance of remnant complications may lead to a different approach to therapeutic decisions. Laparoscopic excision has become the preferred approach in the last decades over laparotomy [2].

We report the case of patent urachus in a 45-year-old man, suspected by the clinical presentation, diagnosed on CT scan, and treated with laparoscopic umbilicus-sparing excision. We will review and discuss the literature on surgical treatment of urachal diseases and related complications.

## Case presentation

A 45-year-old man was referred to the urology service with a history of recurrent urinary tract infections in the last two years and was treated with multiple antibiotic regimens and analgesics. The symptomatology began as mild suprapubic pain associated with clear and fetid serous fluid discharge from the umbilicus that presented with elevations of intraabdominal pressure, dysuria, and occasional hematuria, being the reason for the medical visit. Clinical examination revealed a painful abdomen in the hypogastric region but no signs of peritonitis. No indurated or omphalitis was present on the physical examination. Laboratory tests showed a white blood cell count of 10,000μL, and urinalysis revealed leucocituria and microscopic hematuria without bacteriuria. 

**Figure 1:**
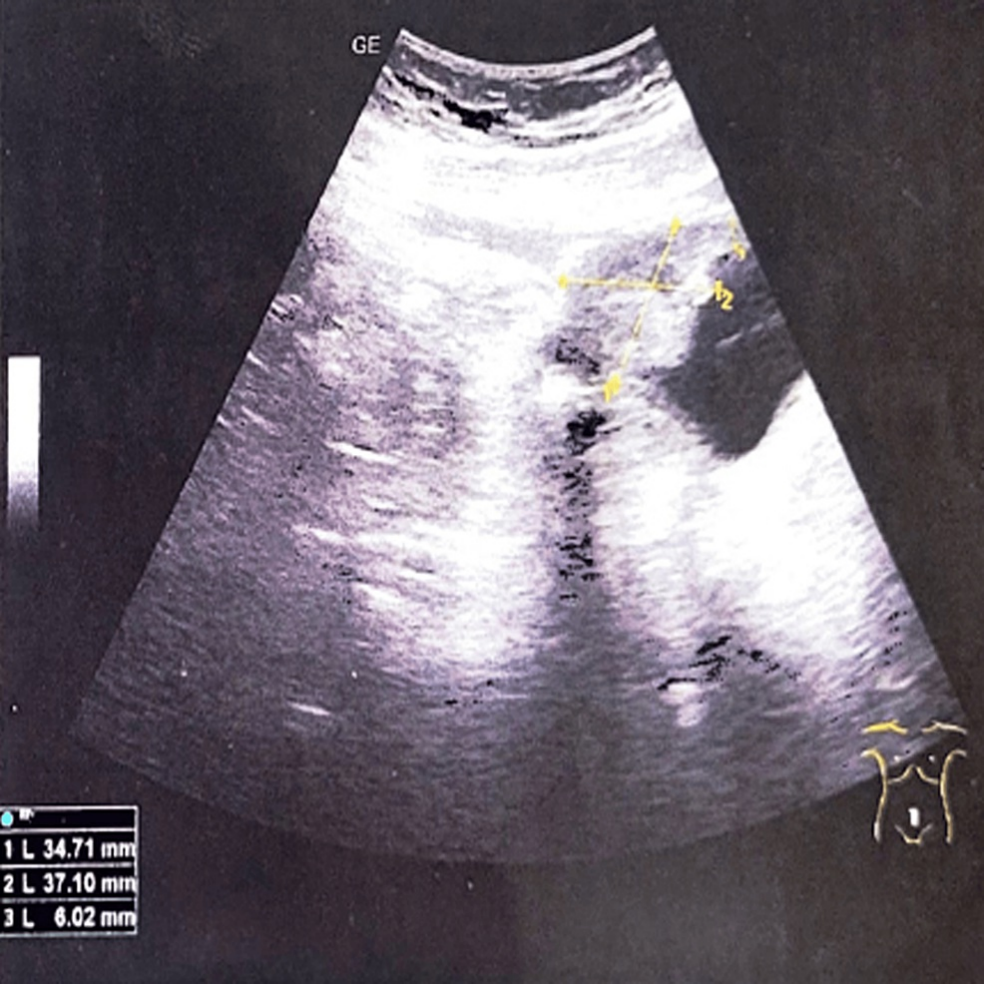
Bladder ultrasonography showing a heterogeneous bladder dome tumor of 34.7*37.1mm measurements, sagittal view

Abdominal ultrasonography reported an anterosuperior bladder wall thickening up to 7 mm. The presence of a solid and heterogenous tumor of the bladder dome of 37.1*34.71 mm, continuous with a permeable urachus. It also showed homogenous bladder content (Figure 1).

**Figure 2:**
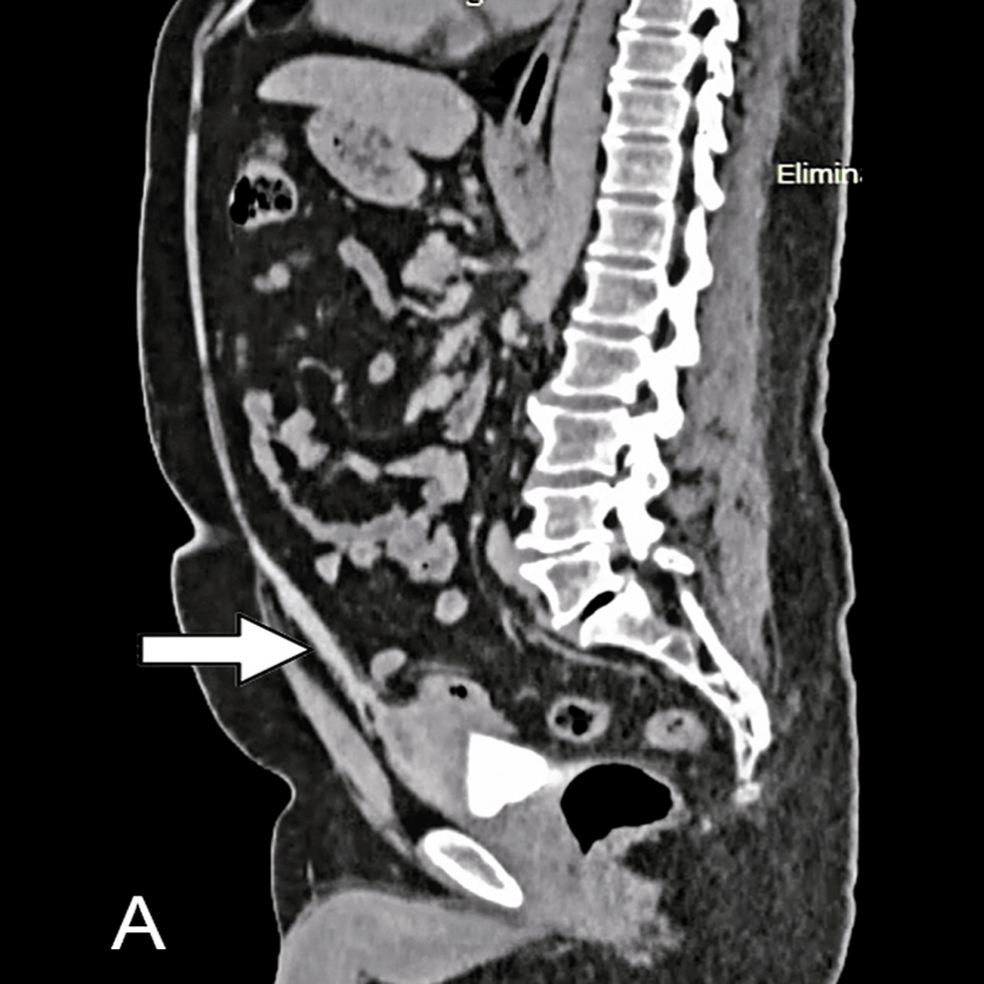
Abdominal enhanced CT scans showing patent urachus (arrow) from the umbilicus to the bladder dome, sagittal view

**Figure 3:**
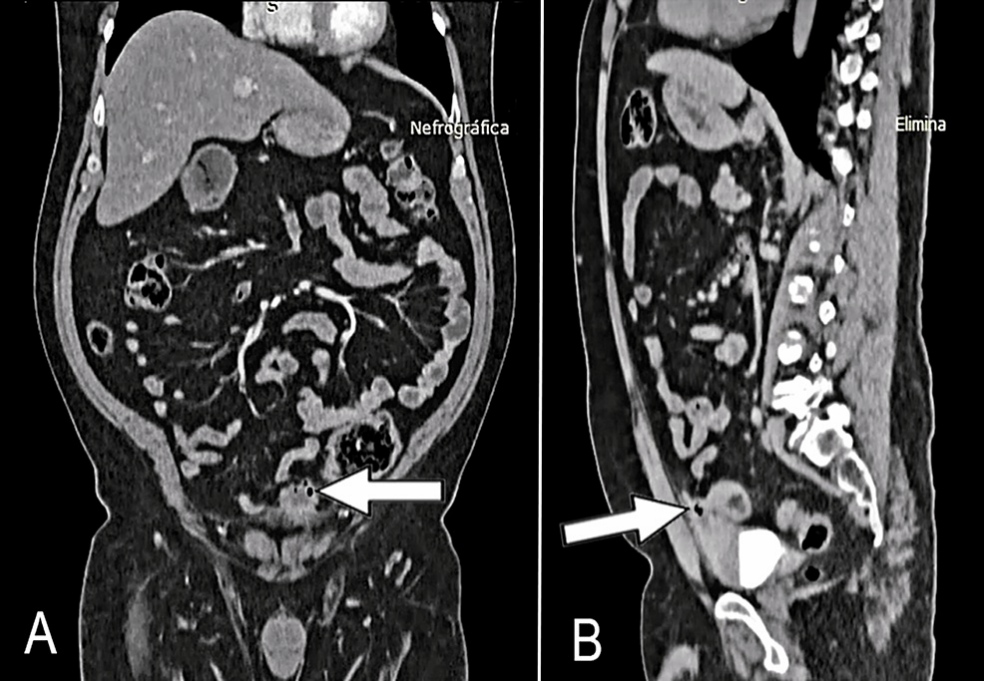
A) Abdominal enhanced CT scan, coronal view, with the presence of heterogeneous tumor in the bladder dome (arrow), with gas in its interior. B) Sagittal view demonstrating continuity with patent urachus

An abdominal contrast-enhanced computed tomography scan elucidated a tubular structure in the Retzius space, possibly a urachus remnant (Figure 2). Additionally, an image with an irregular morphology, well-defined margins, and interior gas was observed in the anterosuperior wall of the bladder, extending towards the lower portion of the urachus. No enhancement following the administration of contrast (Figure 3).

A cystoscopy was performed, which revealed a tumor in the bladder dome approximately 3 cm in diameter without invasion of the bladder mucosae. A biopsy was taken to rule out the presence of malignancy. Histopathology reported the presence of chronic cystitis, urothelial mucosae with chronic inflammatory changes, and granulation tissue. No malignant tissue was reported in the biopsy.

Elective laparoscopic excision of the urachus remnant was performed two weeks later. The patient received general anesthesia and was then placed in a Trendelenburg position to empty the contents out of the pelvis.

**Figure 4:**
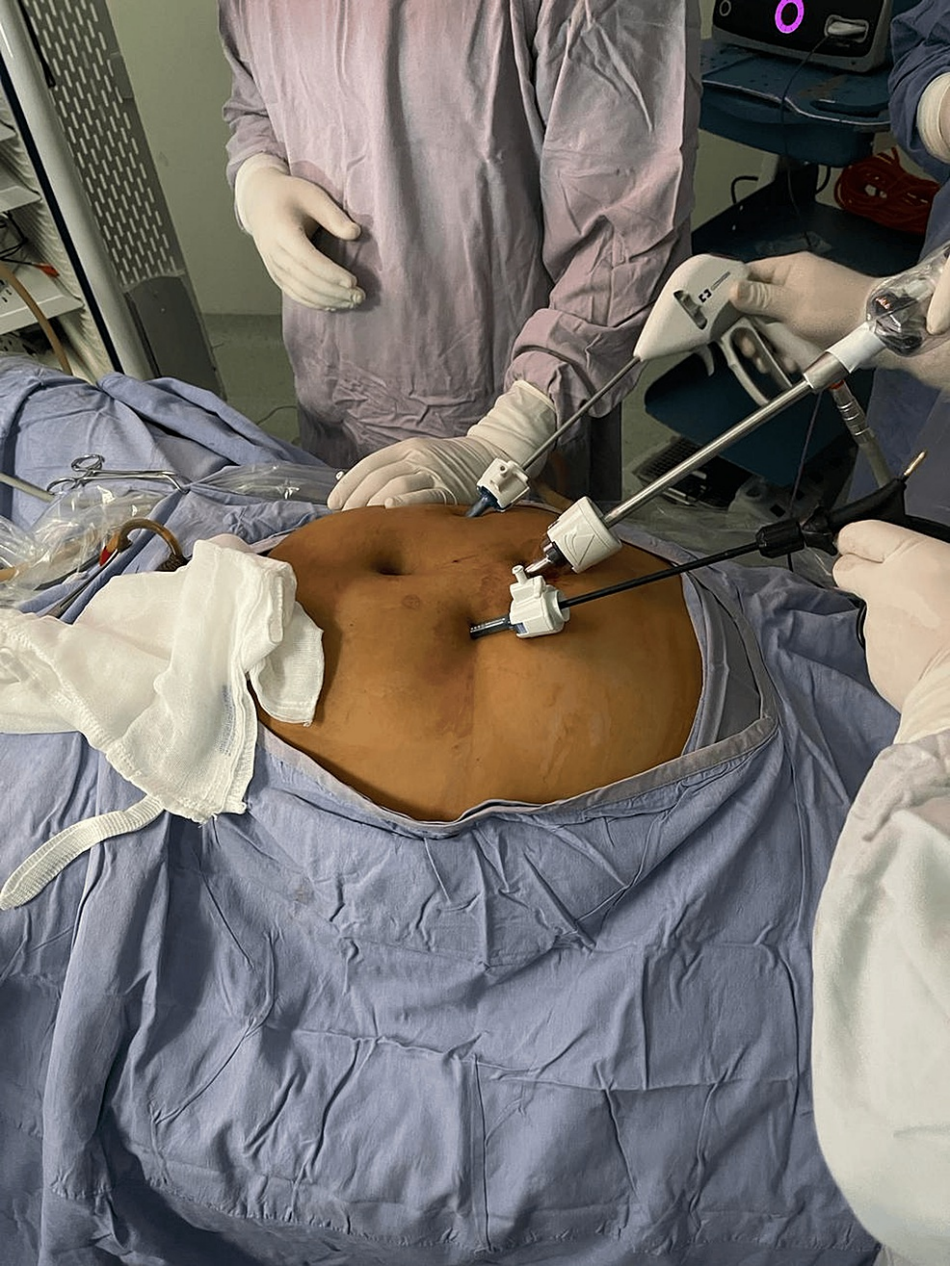
Supraumbilical laparoscopic port placement for removal of the urachal remnant

**Figure 5:**
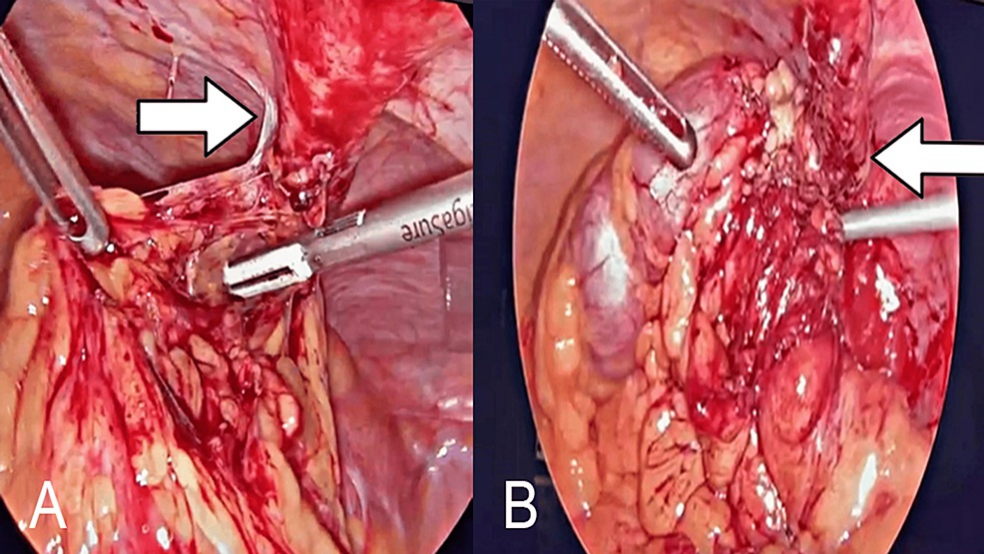
A) Laparoscopic view of patent urachus (arrow) being dissected. B) Plastron attached to bladder dome and urachus (arrow)

**Figure 6:**
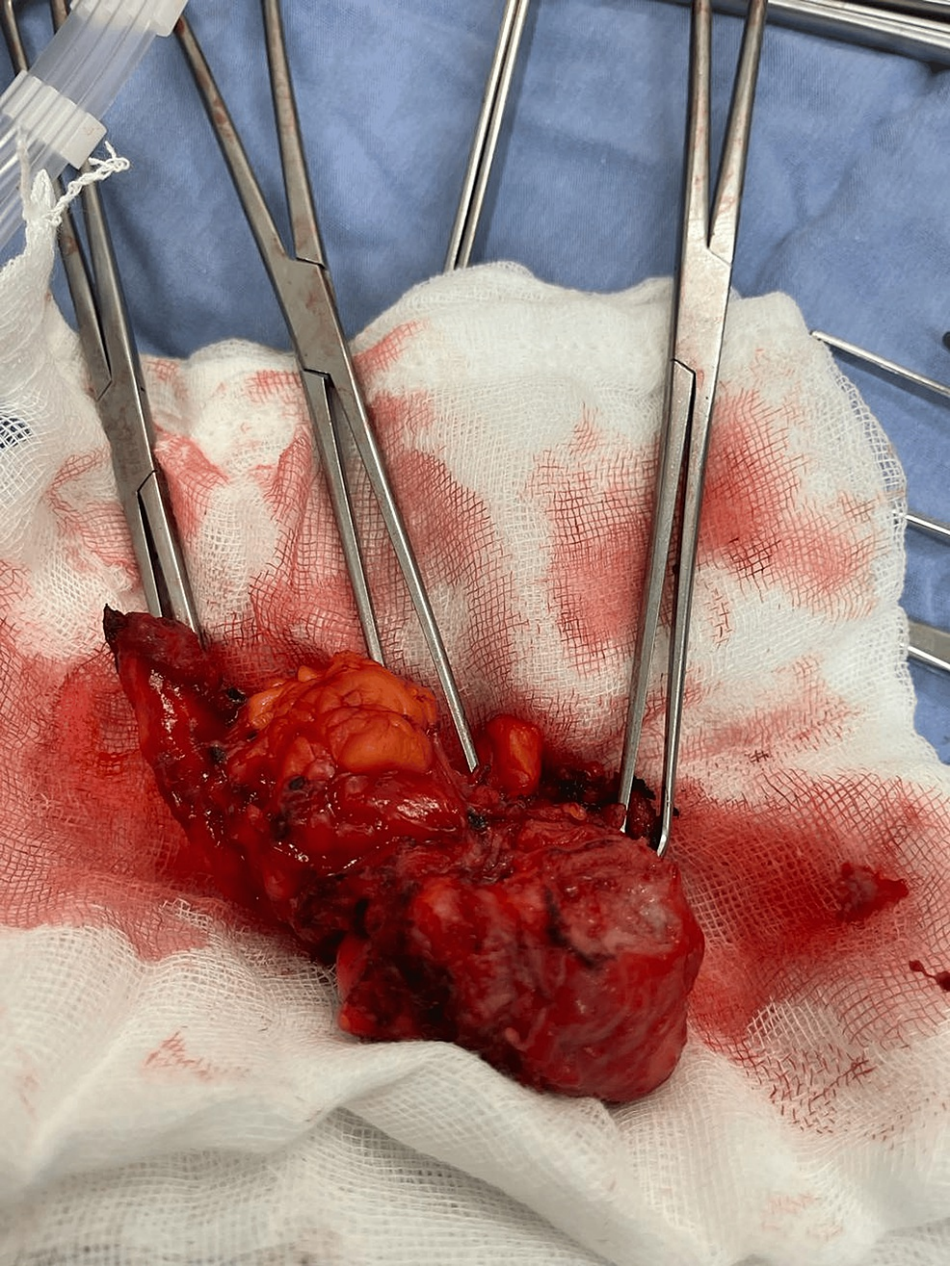
Macroscopic morphology of patent urachus resected

The pneumoperitoneum was created with a Veress needle with supraumbilical insertion. We adopted the three conventional port techniques, involving a 12mm supraumbilical trocar for the 30° laparoscope and bilateral paraumbilical 5mm trocars, allowing for an optimal triangulation and avoiding the umbilicus and potential infected inflamed tissue (Figure 4). The median and lateral umbilicus ligaments were identified, then the entire urachus with wide peritoneal wings was dissected from the transversalis fascia up to the level of the umbilicus and caudally until the space of Retzius where the urachal remnant was inserted into the dome of the bladder. After the entire urachus was dissected free, a plastron was resected from the retropubic space up to the bladder dome; then a partial cystectomy was performed in the anterior bladder dome, including the urachal insertion to resect the complete connection (Figure 5). The bladder defect was laparoscopically closed in two layers, using 4-0 and 1-0 monofilament polydioxanone suture (PDS). The entire specimen was retrieved in an endo-bag and sent for pathological examination (Figure 6). The operating time was 120 min approximately, with a 400 cc blood loss.

The patient had an uneventful postoperative recovery and was discharged after three days of in-hospital stay. The transurethral Foley catheter was withdrawn three weeks later. No symptomatology recurrence during a 12-month follow-up.

The histopathology report confirmed the diagnosis of a patent urachal chord with transitional and columnar epithelia associated with a urachal cyst, inflammatory cell infiltration, and fibrotic tissue in the bladder dome wall, without evidence of malignancy tissue.

## Discussion

While thought uncommon, urachal remnants occur in about 1.6% of children under 15 with an incidence of approximately 1:5,000, and only in 0.063% of adults with an incidence approximately of 1:150,000, with a higher prevalence in males compared to females. In most cases, urachal remnants are asymptomatic and incidentally diagnosed during cross-sectional imaging or a surgical exploration in adulthood [4,5].

These abnormalities are clasified based on where along the urachal path, the abnormal residual patency is found: patent urachus (47%), where the entire tubular structure fails to close leaving an open urachus that connects the bladder and the umbilicus, leading to drain urine from the umbilicus; urachal-umbilical sinus (17%), where the umbilical end of the urachus doesn't obliterate and remain as an evagination below the umbilicus, in which the accumulation of cellular debris can favor complications such as infections or stone formation, with a periodic leakage through the navel and wet umbilicus; urachal cyst (30%), where proximal and distal ends of the urachal canal obliterate resulting in a patent segment along the urachus, usually the distal end of the urachus, in most cases asymptomatic, or becomes symptomatic because of cyst complications; vesicourachal diverticulum (3%), a failed obliteration at the distal end of the urachus, resulting in an outpouching from the bladder dome, being the most rarely detected urachal anomaly with the lower frecuency of complications [4,5]. The most common urachal abnormality in adults is a patent urachus, followed by a urachal cyst, with periumbilical drainage being the most common clinical presentation and infection the most common complication [4,6].

Infections of urachal remnants can cause abdominal pain, abdominal tenderness, omphalitis, fever, nausea, vomiting, dysuria, voiding difficulty, urethritis, epididymitis, and orchitis at presentation, also may predispose patients to other acute complications: cyst rupture, bladder fistula, peritonitis, and sepsis. Furthermore, urachal remnants raise the risk of urachal adenocarcinoma in adults. The estimated incidence is about 0.18 per 100,000 individuals each year, representing 0.1% to 0.3% of all bladder malignancies but accounting for a significant proportion of 20% to 39% of bladder adenocarcinomas [3].

When it comes to diagnosing urachal anomalies, ultrasound is the most commonly used imaging test in children. Computed tomography is more sensitive for diagnosing urachal remnants, identifying complications involving the urinary bladder or neighboring structures, and planning surgical intervention [5]. Its use increases the diagnosis of asymptomatic urachal anomalies during adulthood, providing insight regarding the underdiagnosis of these pathologies being the majority asymptomatic [7]. It may also be of valuable use in diagnosing urachal tumors, especially macroscopic calcifications, which have been reported to be present in 30% of urachal malignant tumors [8].

Initial imaging during the first few months of life may identify asymptomatic remnants, but in up to 80% of patients below six months and 50% of patients 12 months old, these anomalies may be no longer visible because of spontaneous involution. Thus, the surgical resection of asymptomatic anomalies is not indicated in the first year of life [9]. Most should be managed with observation, with infected remnants initially receiving antibiotic treatment and a follow-up ultrasound to monitor for involution. If conservative management fails or presents multiple symptomatic episodes, visible umbilical urine drainage, peritonitis, or abscess formation, then surgical management should be considered [10-12].

In adulthood, the approach to symptomatic remnants differs from children. Surgical resection of embryological remnants has been the gold standard for symptomatic patients. Recently, some have proposed a two-stage management approach with antibiotics for infected remnants before operative excision, emphasizing infection treatment before surgery to reduce the risk of surgical complications, while others prefer first the resection of infected tissue without giving time for infection and inflammation to recede [13]. The conservative option has been shown to effectively treat symptoms and reduce the rate of complications, thereby eliminating the need for urgent surgery. A recurrence of 30% has been reported when complicated urachal remnants are managed conservatively [8].

Surgical excision is the preferred approach to prevent recurrence and posterior complications in persistently symptomatic patients or suspected acute complications [4]. The standard surgery for a symptomatic urachal remnant involves a laparotomy through the midline and infra umbilical incision.

The first laparoscopic surgery was described by Trondsen in 1993 [14]; nowadays, it is considered the gold standard technique for the removal of urachal remnants with faster recovery, better results, and a lower rate of complications. It has been described as an en bloc or wide local resection of the remnant from the proximal to the distal end along with adjacent inflammatory tissue and then removing it via the umbilicus, followed by an umbilicoplasty reconstruction [4,15].

Historically, multiple studies recommend the excision of asymptomatic persistent urachus to lower the risk of future infection and urachus malignancy in adults, given its aggressiveness, late symptom formation, and poor overall survival of less than 50% [7,16]. Gleason et al. discovered that asymptomatic patients with prophylactic resection do not decrease the incidence of malignancy in adults and that the incidence of malignancy is vanishingly minimal. Furthermore, the minimal number to treat with urachal anomalies that would need surgical excision to prevent one single case of malignancy is relatively high, at 5721 remnants [3].

The management given to this patient was based on the laparoscopic umbilicus-sparing approach proposed by Siow et al. [15], where a total and rigorous dissection of the urachus and the inflammatory tissue is performed without an umbilicoplasty, obtaining resolution of symptomatology, achieving infection control, less postoperative pain and superior cosmetic result with no symptomatic recurrence during the follow-up. We realized a bladder cuff excision because an inflammatory plastron was attached to the anterior bladder wall, probably associated with previous infections; furthermore, communication with the bladder was suspected. Up to date, a consensus for the resection of a bladder cuff does not exist; some authors recommend this as a routine step to prevent recurrences and carcinoma occurrence. If there is a possibility for residual tissue, we recommend the excision of the bladder cuff only when an adherent and communicating remnant attached to the bladder diagnosed by imaging, cystoscopy, and during surgery; moreover, in cases with unfavorable histology from cystoscopy that suggests malignancy, as transitional epithelium, squamous metaplasia, intestinal metaplasia, or mixed metaplasia [17]. No previous studies have reported umbilical recurrence in cases with carcinoma in which umbilicus-sparing was performed during partial or total cystectomy. Umbilical locally advanced urachal tumors must be resected totally, ruling out the possibility of performing an umbilical-sparing technique [18].

## Conclusions

Although believed to be rare, urachal remnants incidence is higher than reported in the past, with an increased diagnosis during routine imaging, leading to the identification of incidental cases. Early detection may be a benefit for the improvement of the treatment approach and preoperative planning, aiming for total resection of the urachus when conservative treatment has failed. A lack of randomized controlled trials and prospective studies, associated with the rarity and scarcity of existing data, makes us unable to determine the ideal consensus of a preferred regimen. Laparoscopic umbilicus-sparing excision seems to be a safe and minimally invasive method for the treatment of urachal remnants, earning recurrence control, less symptomatology, and pain recurrence than umbilicoplasty in the postoperative follow-up, with superior cosmetic results. A prospective large-scale, multicenter randomized study analyzing demographic information, urachal anomalies subtypes, and symptomatology needs to be performed to identify the most effective treatment approach.
